# Study of the Incorporation of Biomass Bottom Ashes in Ceramic Materials for the Manufacture of Bricks and Evaluation of Their Leachates

**DOI:** 10.3390/ma13092099

**Published:** 2020-05-01

**Authors:** Juan María Terrones-Saeta, Jorge Suárez-Macías, Francisco Javier Iglesias-Godino, Francisco Antonio Corpas-Iglesias

**Affiliations:** Department of Chemical, Environmental, and Materials Engineering, Higher Polytechnic School of Linares, University of Jaen, 23700 Jaen, Spain; jsuarez@ujaen.es (J.S.-M.); figodino@ujaen.es (F.J.I.-G.); facorpas@ujaen.es (F.A.C.-I.)

**Keywords:** ceramic, brick, biomass bottom ash, clay, compression resistance, leachate, environment, circular economy, sustainability

## Abstract

Scarcity of raw materials, reduction of greenhouse gas emissions and reduction of waste disposal in landfills are leading to the development of more sustainable building materials. Based on these lines, this work studies the incorporation of biomass bottom ashes into ceramic materials for brick manufacture, in order to reuse this currently unused waste and reduce clay extraction operations. To this end, different groups of samples were made with different combinations of clay and biomass bottom ashes, from 100% clay to 100% biomass bottom ashes. These samples were shaped, sintered and subjected to the usual physical tests in ceramics. In turn, the mechanical resistance, color and leaching of the contaminating elements present were studied. The physical and mechanical tests showed that the results of all the families were adequate, achieving compressive strengths of over 20 MPa and leaching of the contaminating elements acceptable by the regulations. Therefore, a sustainable range of ceramics was developed, with specific properties (porosity, density, resistance and color), with a waste that is currently unused and sustainable with the environment.

## 1. Introduction

The construction sector is one of the essential sectors for the development and well-being of a population. However, this sector is also highly polluting, as it consumes large quantities of virgin materials for the production of materials [1]. In turn, it also generates large emissions of greenhouse gases in the manufacture of materials, cement, ceramics, bituminous mixtures, composite materials, etc. [2]

Within the construction sector, building is very important. Constructing buildings for a growing population or renovating old buildings is essential for the comfort of the population. More specifically, ceramic materials account for a high percentage of the materials used in buildings. In turn, bricks are the most widely used ceramic material.

Red clay bricks have been produced for thousands of years. Their versatility, economy, resistance and good behavior make them an essential material in building, so much so that the world’s production of bricks is approximately 1400 billion units. To manufacture these bricks, it is essential to extract clay, a material that may become scarce in the future [3,4,5]. This means that major countries, such as China, are limiting their production [6,7]. However, this is not the best solution, since such an important sector as the building industry cannot be stopped. Today there are new trends to reduce consumption of virgin materials or optimize processes [8]. These trends include the development of geopolymers or the incorporation of waste into bricks.

The incorporation of waste is a good solution. On the one hand, an unused waste is used and not deposited into a landfill. On the other hand, the cost of the final product, brick, is cheaper. Furthermore, in most cases, this waste can be incorporated without major changes to the equipment or process [9]. The incorporation of waste into new materials makes it possible to create sustainable materials in the context of the circular economy [10].

Today, there is a lot of waste and of different categories. It is essential to study the physical, chemical and mechanical properties of the waste for its correct incorporation. With the detailed study of the waste, it is possible to incorporate these wastes with demonstrated viability [11].

In turn, these wastes can provide the final material with specific properties. Among these properties are resistance, color or thermal and acoustic insulation. For example, the incorporation of pore-forming wastes makes the final material less dense, more porous and, consequently, less thermally and acoustically conductive.

Different authors have studied the incorporation of waste in the brick-manufacturing ceramic industry. The wastes incorporated were chamotte [12,13,14,15], ashes [16,17,18,19,20], sludge from ornamental stone cutting [21,22,23,24,25,26,27,28], construction and demolition waste materials [29], water-treatment-plant sludge [30,31,32,33], glass waste [34,35], mold flux waste [36,37,38], steel sludge [39,40,41], etc. In these different works, the characteristics of the waste were first studied in order to evaluate the maximum acceptable level of the waste in the final material.

In this work, the incorporation of biomass bottom ash (BBA) from the combustion of olive pruning and vegetable fat wastes for energy generation is presented. This waste is currently a problem, as there is no use for this material and it is landfilled. This waste is classified as non-hazardous; however, it has high percentages of some chemical elements that can present environmental problems. Therefore, the use of these biomass bottom ashes has a triple function: firstly, to eliminate the deposition of this waste in a landfill; secondly, to obtain ceramic material, brick, cheaper; and thirdly, to retain the chemical elements that may cause damage to the environment [42].

It should be borne in mind that world production of biomass is approximately 140 billion tons per year [43], so the production of biomass ash is very high. Biomass bottom ash is a by-product of biomass combustion. This product has both inorganic and organic chemical elements [44], the latter being due to incomplete combustion of the biomass due to high temperatures and short exposure time. The chemical composition of the biomass bottom ash is strongly influenced by the biomass used [45], so each case must be studied individually. However, in this case the chemical composition is maintained over time, as the biomass used is always the same. This fact is very important for the use of a waste, i.e., that the chemical composition remains as unaltered as possible over time. This is not the case for other wastes and is a problem for the manufacture of new materials containing them, for example water treatment sludge, municipal solid waste ashes or construction and demolition waste.

Although biomass bottom ash (BBA) is a good waste for use, most companies in different countries prefer to deposit it in landfills [46]. There are some cases where biomass fly ash (BFA) has been used in different products [47,48,49]. However, biomass bottom ash has had much less study and use. There is work in which these biomass bottom ashes are incorporated in construction as an additive to cement [50]; however, they showed a reduction in compressive strength in their replacement [51,52].

However, it should be remembered that the chemical composition of biomass bottom ash varies greatly depending on the biomass used for combustion. Therefore, biomass bottom ash containing percentages of sulfur and chlorine, which are quite common, can cause the cement produced to have significant resistance problems, as well as in contact with steel. The usual zinc oxide in some types of biomass bottom ashes generates problems in the setting of the cement; in addition, it disperses randomly, so it is a very restrictive compound for its use in cements. In short, the use of biomass bottom ashes as a partial substitute in cements is restricted to low percentages of substitution and not to all types of ashes, causing the benefits (economic and environmental) to be less than the problems that can arise if a detailed study of its chemical composition is not carried out.

In addition, new studies have emerged in which biomass bottom ash is used as a stabilizing element in road soils with expansion problems [53], for example, marls. Marl is a type of soil composed of limestone and clay in different proportions that does not have sufficient strength as a sub-base for the road and can also cause problems of expansiveness in contact with water. Traditionally, a treatment has been carried out with lime first and then with cement, to avoid the problems mentioned. This double treatment implies an important cost in the construction of the road due to the volumes of lime and cement used and the large dimensions of the infrastructure. As a solution to this problem, there are studies in which a stabilization with biomass bottom ash is carried out for this type of soil. Biomass bottom ashes contain percentages of silicon oxide and calcium oxide, so they can perform this double treatment with acceptable results. Furthermore, road construction consumes a large amount of this waste, given the size of these infrastructures. However, this technique has two fundamental problems; on the one hand, it is usual for the biomass bottom ashes to be mixed with water in the production industry, to avoid combustion problems, thus causing inactivity of the silicon and calcium oxides when in contact with water. On the other hand, the composition of biomass bottom ashes, as commented, varies greatly depending on the type of biomass. It is usual to find in its composition elements such as sulfur, chlorine or heavy metals. Therefore, their use in these large infrastructures exposed to the weather and water can cause the leaching of these pollutants to surface water and groundwater. Furthermore, road infrastructures do not have the waterproofing that landfills have for leachate retention. Therefore, their use in road infrastructures without the exhaustive control of the polluting elements and the leachates produced can create more negative effects than the deposition of biomass bottom ashes in a landfill.

The use of biomass bottom ash in ceramics for brick production has several advantages over detailed studies. Firstly, the production of bricks is high, so the use of the ashes avoids the extraction of clay as virgin material (with the consequent environmental impact and greenhouse gas emissions) and is able to use significant quantities of this biomass bottom ash. Secondly, landfill deposition and the consequent likely contamination of surface and groundwater by the chemical composition of the ash is avoided. Thirdly, these pollutants are retained in the ceramic matrix, avoiding environmental contamination, and these results are evaluated with leachate tests. Fourthly, particular properties are achieved for the ceramics formed with the biomass bottom ashes, such as a lower density, creating a lighter material; a higher porosity, creating a material with a probable lower thermal and acoustic conductivity; and a wide range of colors, making the ceramics different from the traditional ceramic. However, care must be taken in production, to ensure that these characteristics are maintained, that the chemical composition of the biomass bottom ash is invariable, that the values established by the regulations are met and that the leachate produced is acceptable.

Based on the above, the present work studies the incorporation of biomass bottom ashes from the combustion of olive pruning and vegetable fat wastes in ceramic materials, bricks. The residue was analyzed, and different samples were conformed with different percentages of the residue. These samples were studied with physical and mechanical tests, to obtain the maximum incorporation. The existence in the biomass bottom ashes of polluting elements that could be hazardous to the environment made it necessary to carry out leachate studies to study the retention of these elements into the ceramic matrix.

All families of ceramics made from clay and biomass bottom ash showed acceptable physical properties. In turn, resistance decreased with the increase in the percentage of biomass bottom ashes added; however, in all cases, it was higher than the minimum required by European regulations for this type of material. The leachate tests showed a retention of the contaminating elements, obtaining results below the maximums marked by the regulations. Therefore, a range of ceramics with different percentages of addition of biomass bottom ashes acceptable for use and with a wide range of colors recorded in the colorimetric tests were obtained.

## 2. Materials and Methods

### 2.1. Materials

The materials used in this work were mainly red clay and biomass bottom ashes. Both elements are studied in the Methodology section; however, their general characteristics and origin are described in the following sections.

#### 2.1.1. Clay

For the performance of this project, we used red clay from the province of Jaen, Spain. This clay is usually used for the manufacture of bricks by companies in the abovementioned area.

The red clay has been used as a base material for the incorporation of biomass bottom ashes in different percentages. This material has a good granulometry and suitable characteristics for brick manufacture. The subsequent physical and chemical tests carried out have been able to corroborate this fact.

#### 2.1.2. Biomass Bottom Ash

The industry of electricity generation through the combustion of biomass produces a series of industrial by-products. These wastes have different chemical and physical characteristics, depending on the biomass used and the process. Among these residues are biomass bottom ashes (hereinafter referred to as BBA), which must be studied individually for their use.

In this work, the biomass bottom ashes used come from the combustion of olive-pruning remains and vegetable-fat wastes for the generation of electric energy. The producing companies are located in the province of Jaen, Spain, and use as biomass the typical residues of the nearby industries. These biomass bottom ashes have particular physical and chemical characteristics, not suitable for use until now. 

These biomass bottom ashes have a granulometry of less than 6 mm, being easily crushed to achieve a smaller size and make them compatible for mixing with the clay. The biomass bottom ash was physically and chemically characterized to evaluate its suitability for brick manufacture, as detailed in the Methodology section.

### 2.2. Methodology

The methodology followed in this work has a series of logically ordered tests to reflect clear and objective results on the viability of incorporating biomass bottom ash into ceramics for brick manufacture. At the same time, the chemical elements pollutants of the biomass bottom ashes will be retained in the ceramic matrix.

Firstly, the materials used in the conformation of the ceramics were analyzed to evaluate their suitability. To this end, physical and chemical tests were carried out on both materials. The physical tests were the calculation of the particle density and the plasticity index. These tests are essential to characterize the raw materials for ceramics. On the other hand, chemical tests were carried out to find out the composition of both products. These chemical tests were elemental analysis, loss on ignition and X-ray fluorescence. It should be noted that the biomass bottom ashes from the combustion of the olive-tree pruning and vegetable-fat wastes has a granulometry lower than 6 mm; therefore, it was ground to obtain a granulometry lower than 0.25 mm. This granulometry is compatible with red clay and facilitates mixing operations in the different percentages.

Subsequently, different families of ceramic material samples were conformed with different percentages of clay and biomass bottom ashes. To this end, a group of samples without the addition of ashes and with only clay was used to start, and then increasing percentages of 10% biomass bottom ash were added, obtaining different groups of samples. The last group was composed only of biomass bottom ash without clay. In this way, it was possible to evaluate objectively, and after carrying out the subsequent physical, mechanical, aesthetic and leachate tests, the variation in the properties of the ceramic with the addition of biomass bottom ash.

A steel matrix and a compaction pressure of 30 MPa were used to conform the samples. The samples obtained were sintered in an oven at a temperature of 950 ± 10 °C, after drying at 105 ± 2 °C for 24 h. The physical tests carried out on the groups of manufactured samples determined the effect of the incorporation of the biomass bottom ash into the resulting material. In turn, the color of the different families was evaluated objectively, using a laboratory instrument called a colorimeter.

Once the physical properties were evaluated, the mechanical resistance of the different families of samples was determined with the compressive strength test. With the limits of the current regulations regarding resistance, the maximum percentage of biomass bottom ash addition was studied.

Finally, and after carrying out the different tests, the retention of the chemical element pollutants in the biomass bottom ashes in the ceramic matrix was evaluated. For this purpose, the leachates in water from the different families of samples were analyzed and compared with the leachates from the unaltered biomass bottom ash sample.

On the basis of the above, the subsequent sections were divided into three large groups of tests. First, the initial materials are characterized; then the samples are conformed, and the physical, mechanical and colorimetric tests are carried out; and, finally, the leachates of the different families are studied.

#### 2.2.1. Physical and Chemical Characterization of Materials

In this section, we discuss the appropriate physical and chemical tests that were carried out to evaluate the suitability of the clay and biomass bottom ashes for the manufacture of ceramics. It should be noted that the materials were crushed, then dried at a temperature of 105 ± 2 °C for 24 h and sieved through a 0.25 mm sieve. This process was carried out in order to have control of all the parameters. However, the existence in industry of humidity would not harm the final material; it should simply be taken into account so as not to add excess water. At the same time, sieving both materials until obtaining a maximum size of 0.25 mm is to homogenize them and to facilitate their mixing.

The tests carried out can be classified into two groups: firstly, the physical tests to determine the particle density (UNE-EN 1097-7 standard) and the plasticity index (standards UNE 103103 and UNE 103104); and secondly, the chemical tests to determine the chemical elements in the samples, elemental analysis, loss on ignition and X-ray fluorescence. 

The calculation of the particle density for the clay and biomass bottom ashes was done with the pycnometer, determining with water and successive volume and mass measurements the particle density of both materials. The plasticity index, essential for clayey materials, was found by the Casagrande method, using mainly the Casagrande ladle for the calculation of the liquid limit and the respective procedure for the calculation of the plastic limit. The difference between both values reflected the plasticity index.

The elemental analysis automatically measures the gases, for the detection of carbon, hydrogen, nitrogen and sulfur, produced in the complete combustion of a sample. This test was performed with LECO’s TruSpec Micro commercial equipment (TruSpec Micro, LECO, St. Joseph, MI, United States). In turn, the loss on ignition determines the variation in mass when the sample is subjected to the temperature of 1000 ± 10 °C, a very relevant datum for the incorporation of materials in ceramics, since this is the approximate sintering temperature. The X-ray fluorescence was performed with the commercial equipment ADVANT’XP+ (ADVANT’XP+, Thermo Fisher, Waltham, MA, United States), determining the inorganic chemical composition of the majority elements, as well as some elements in smaller proportion.

It should be noted that the study of the chemical elements is fundamental to know which elements can damage the final ceramic formed with a combination of both materials, as well as to recognize the contaminating elements present in the biomass bottom ash. The retention of these contaminating elements in the ceramic matrix are subsequently studied with the leachate tests.

#### 2.2.2. Conformed of Samples: Physical, Mechanical and Colorimetric Tests to Samples’ Families

Once the clay and biomass bottom ashes had been prepared according to the procedure described, and the suitability of the previous tests for the conformation of ceramic materials had been assessed, the different families of samples were conformed.

To this end, clay was taken as the base material, and samples were conformed with 100% of this material (0A10C). The subsequent groups of samples were made with increasing percentages of biomass bottom ashes of 10%, until the last family formed only with biomass bottom ashes was reached (10A0C). In this way, all possible combinations of both materials are developed and the differences in the properties of the final material with respect to the addition of biomass bottom ash can be evaluated.

The families of samples that have been manufactured in this work are detailed in Table 1. In Table 1, the name of the family, the percentage of clay and the percentage of biomass bottom ash can be identified.

Six samples from each family were made by compression in a steel matrix. This matrix has internal dimensions of 60 × 30 mm. The mixture of the two materials was poured into the matrix and subsequently compacted by means of a 30 MPa pressure. Shimadzu AG-300kNX (AG-300kNX, Shimadzu, Kyoto, Japan) commercial equipment was used to conform the samples with the detailed pressure, compacting the samples at a specified speed and maintaining the maximum pressure for 1 min. The different families of samples were conformed with 10% water, to ensure that the samples were properly formed and were as strong as possible. This percentage of water is empirically determined, with higher percentages causing water exudation during compaction and lower percentages causing lower density.

The conforming process begins with the dry mixing of both materials, clay and biomass bottom ash, according to the corresponding percentage of each family. Subsequently, 10% water is added and mixed again, until a homogeneous material is obtained. This material is then poured into the matrix and compacted as previously indicated.The conformed samples of the different families were removed from the matrix after compaction. Once this process was completed, they were dried at a temperature of 105 ± 2 °C for 24 h, subsequently measuring the geometric dimensions and dry weight of each of the samples.

For the sintering of the ceramic materials, the different samples were introduced into the muffle furnace, and the temperature was raised by 4 degrees Celsius per minute to 950 ± 10 °C. This temperature was maintained for one hour so that, after allowing the samples to cool, their geometric dimensions and weight after sintering could be measured again.

Once the ceramic materials of the different families had been obtained, the following tests were performed: determination of weight loss, determination of linear shrinkage (UNE-EN 772-16 standard), capillary water absorption (UNE-EN 772-11 standard), cold-water absorption (UNE-EN 772-21 standard), boiling-water absorption (UNE-EN 772-7 standard), bulk density and open porosity (UNE-EN 772-4 standard).

The determination of weight loss consists of the study of the variation of the mass before and after sintering, thus evaluating the variations caused by the incorporation of the waste that will condition the physical and mechanical properties of the final material. Capillary water absorption is an essential parameter for ceramic materials that are outdoors. The test consists of calculating water absorption when the sample is partially submerged in water in a very short time. On the other hand, and in line with the previous test, the absorption of boiling water and cold water consists of the study of the variation of the mass after immersing the samples in boiling water or cold water (as appropriate) for a long time. The ratio of the increase in mass to the dry mass will represent the results of both tests under different conditions. The calculation of bulk density and open porosity is carried out by using relationships laid down in the standard, in which the initial data are the dry mass, the mass immersed in water and the mass with water absorption of the test pieces. The use of precision hydrostatic balances is essential for the calculation of these masses.

Once the previous tests were carried out, we determined the compressive strength (UNE-EN 772-1 standard) of the different families of samples. This test is essential for a ceramic, according to the regulations on the subject. The families of samples that develop resistance lower than that established by the standards will be discarded. The test was carried out with the detailed equipment controlling the test speed and the breaking strength. The samples of all families for this test must be dry, not containing humidity that could alter the results. Finally, and as mentioned above, it is necessary for the marketing of a ceramic material that has a pleasant visual appearance. This property is difficult to evaluate, since the market is directly the one that decides the acceptance of a product. However, to objectively determine the color of the material, the colorimetry test is carried out with the instrument designed for this purpose called a colorimeter. The colorimeter used for this test was the PCE-RGB 2 model (RGB-2, PCE, Meschede, Germany). The samples of each family were polished on the surface, to reflect the internal and real color of the material, and then the color was analyzed with the colorimeter after calibration with the white color. This measurement provides the Red, Green and Blue coordinates of the color of the samples, so as to faithfully reproduce the color of each one of the families by graphic means. The color is another characteristic of the different groups of samples that will greatly condition the viability of the product’s commercialization, so this property should not be underestimated.

#### 2.2.3. Leaching Tests of the Different Families of Samples

One of the main objectives of the present work is the retention in the ceramic matrix of the chemical elements’ pollutants existing in the biomass bottom ashes. For this purpose, the most suitable test with an excellent sensitivity is the leaching test. The leaching of unaltered biomass bottom ashes (taken directly from the producing industry) has been compared with the different families of ceramics conformed with the addition of the same. In this way, the retention of chemical elements’ pollutants in the ceramic matrix can be evaluated. The process summarized according to the UNE-EN 772-5 standard is described below:

- The dry, milled sample to be leached was taken and was weighed at about 20 ± 0.05 g.
- It was then poured into a polyethylene bottle with 200 mL of deionized water.
- It was then shaken for 60 ± 2 min by means of a horizontal shaker at 120 ± 5 min-1, with a horizontal movement of 20 mm and room temperature.
- After 15 ± 1 min from the completion of the extraction, the sample in suspension was filtered, using an ash-free blue-band paper filter, and the filtrate was collected in a clean flask.

Once the leachate samples have been taken for the different families of ceramics formed, as well as the sample of biomass bottom ash, we proceeded to analyze with Inductively Coupled Plasma Mass Spectrometry. The ICP-MS Instrument is a complex piece of equipment that provides the existing chemical elements and their proportion in the sample under study, thanks to a series of patterns with which the concentration can be obtained. The equipment used is the Agilent 7900 commercial model (7900, Agilent, Santa Clara, CA, US).

The results obtained are later compared between them, to certify the retention in the ceramic matrix. They are also compared with the maximums established by the regulations for the different chemical elements in ceramics. In this way, those families that do not comply with the established leaching parameters can be rejected.

## 3. Results and Discussions

The following sections describe the results obtained from the tests mentioned in the methodology. The results show partial conclusions that allow us to evaluate the feasibility of incorporating biomass bottom ash into ceramic materials for brick manufacture.

### 3.1. Physical and Chemical Characterization of Materials

Once the samples were crushed and dried, they were sieved through the 0.25 mm sieve. The sieved samples of both materials, clay and biomass bottom ashes, were used in all the methodology.

The particle density (UNE-EN 1097-7 standard) calculated for the clay and biomass bottom ashes showed values of 2.44 and 2.85 g/cm^3^, respectively. Both values are very close to each other; therefore, their mixing did not have problems, nor was it necessary to make volume corrections. The density of 2.85 g/cm^3^ for biomass bottom ash was similar to that of a virgin material, so its mass dosage was correct.

On the other hand, the values of plasticity for biomass bottom ashes were zero. This fact is due to its chemical composition, in which there are percentages of cementitious elements, as was seen in later tests. The clay reflected the usual and correct values of plasticity for brick-making. The results of the calculation of the plasticity index (standards UNE 103103 and UNE 103104) are shown in Table 2.

Among the chemical tests performed on biomass bottom ashes and in the clay, elemental analysis is essential. Through this test, the proportion of elements of lower atomic weight, such as carbon, can be quantified. Table 3 shows the results for clay and biomass bottom ash.

Elemental analysis of the clay shows a rather low carbon percentage due to its composition of mainly aluminum silicates. High percentages of carbonates could create problems in the manufacture of bricks, so this clay is acceptable for use. The biomass bottom ash also shows a low percentage of carbon, which is to be expected due to its formation process. The high temperatures of biomass incineration make this possible; however, there is always some percentage of it that is unburned. It should be noted that the carbon detected in this test would correspond to organic matter and carbonates in the material, and it is not possible to distinguish between them by this method.

The loss on the ignition test reflects the loss of weight at high temperatures, 1000 ± 10 °C. This loss of mass may be due to the elimination of organic matter, the elimination of carbonates, the transformation of some chemical compounds or the oxidation of some chemical elements. The results of the loss on ignition of the clay and bottom ashes of biomass are shown in Table 4.

As can be seen in Table 4, the loss on ignition of the clay is relatively low and is mainly due to the transformation of some chemical compounds. On the other hand, the loss on ignition of biomass bottom ash is higher than that of clay but similar. This loss on ignition may be mainly due to the percentage of unburned material created in the incineration process, as well as the transformation of some chemical compounds. As in the elemental analysis test, it is difficult to determine exactly what this percentage of weight loss is due to; however, the final value obtained for both materials is correct for use in brick manufacture.

The X-ray fluorescence for the sample of clay and biomass bottom ash is reflected in Table 5. As you can see, the clay has a main composition of aluminum silicates, with low percentages of other oxides, such as calcium oxide or magnesium oxides. This is an ideal chemical composition for the use of this clay in the manufacture of bricks. On the other hand, the biomass bottom ash reflects a significant percentage of silicon oxide and calcium oxide. These compounds result from the process of biomass bottom ash formation and the biomass used. Potassium oxide is also a common element in biomass bottom ash, being in this case lower than in other types of ash. Magnesium oxide and calcium oxide must be monitored in the ceramic manufacturing process so that they do not harm the final ceramic. On the other hand, there are chemical elements such as sulfur, aluminum, chromium, zinc, strontium, chlorine, copper, manganese, arsenic and nickel that appear in lower percentages but can be very harmful to the environment if they are not correctly retained in the ceramic matrix. These chemical elements were studied by means of Mass Spectrometry after leaching the sample of biomass bottom ashes and the ceramics manufactured with them.

Physical and chemical testing of the clay showed it to be suitable for brick-making, as did the biomass bottom ash. Density compatibility and similarity of size facilitated the mixing of both materials. However, biomass bottom ash has some chemical elements that must be controlled in the process, as well as by leaching, in order not to damage the final product.

### 3.2. Physical, Mechanical and Colorimetric Tests to Samples Families

**Figure 1:**
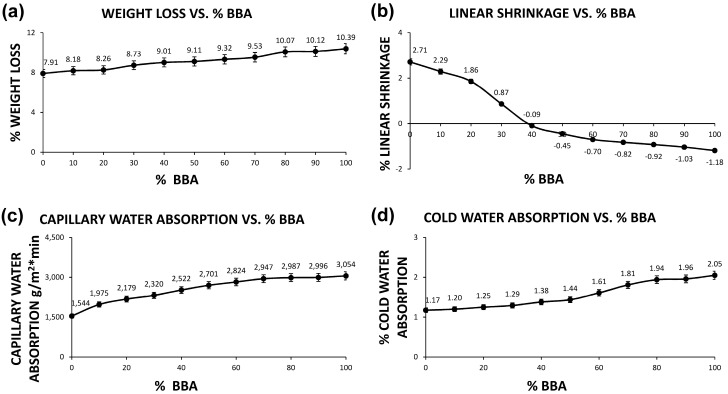
Graphs of the different physical properties of ceramics as a function of the percentage of biomass bottom ash: (**a**) weight loss, (**b**) linear shrinkage, (**c**) capillary water absorption and (**d**) cold water absorption.

The samples formed with the combination of clay and biomass bottom ash, detailed in Table 1, and later sintered at the temperature of 950 ± 10 °C, are analyzed in this section, with different physical tests. The physical tests carried out on the different samples in each group were determination of weight loss, determination of linear shrinkage (UNE-EN 772-16 standard), capillary water absorption (UNE-EN 772-11 standard) and cold-water absorption (UNE-EN 772-21 standard). The results of these tests are shown in Figure 1.

In Figure 1, it can be seen how the loss of weight after sintering is very similar between the different families. This fact is due to the similarity in the loss on ignition between the clay and the biomass bottom ashes. Therefore, the substitution in different percentages of the clay by the biomass bottom ashes does not cause an important variation in the weight loss of the shaped samples. 

The linear shrinkage of the samples decreases with the increase of the percentage of biomass bottom ash. For samples with 40% biomass bottom ash and 60% clay, linear shrinkage is zero, while higher percentages of biomass bottom ash addition cause expansion. This fact is not negative, since the linear expansion percentages are lower than the linear contraction percentages of a traditional ceramic (0A10C), and in none of the cases are they higher than 8%.

The capillary water absorption of the different families increases with the percentage of addition of biomass bottom ash. This fact implies a higher porosity, a lower density and a lower resistance. However, the values are not extreme and are not higher than those marked by the regulations. A higher percentage of suction would imply a greater weight of the manufactured brick if it were exposed to the weather, a fact that should be taken into account for roof tiles and bricks for façades.

The cold-water absorption increases with the percentage of added biomass bottom ash. As in the previous case, this parameter indicates a higher percentage of porosity, which results in a lower percentage of bulk density and, ultimately, lower compressive strength. The values of this parameter are acceptable when waste is incorporated and may provide special characteristics.

**Figure 2:**
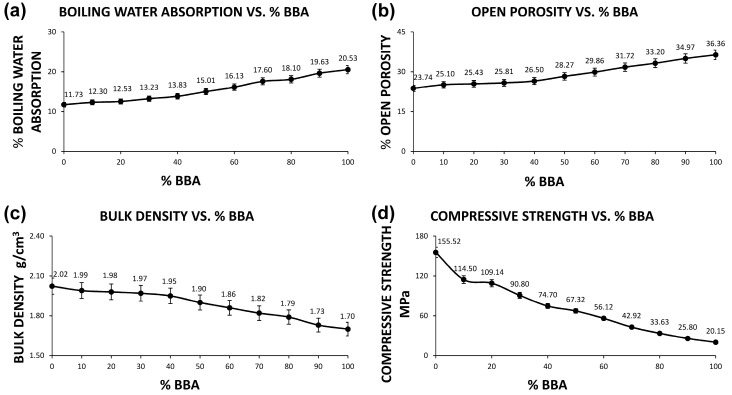
Graphs of the different physical and mechanical properties of ceramics as a function of the percentage of biomass bottom ash: (**a**) boiling water absorption, (**b**) open porosity, (**c**) bulk density and (**d**) compressive strength.

On the other hand, the physical tests of boiling water absorption (UNE-EN 772-7 standard), bulk density (UNE-EN 772-4 standard), open porosity (UNE-EN 772-4 standard) and compressive strength (UNE-EN 772-1 standard) of the different families of samples formed are shown in Figure 2.

As can be seen in Figure 2, boiling-water absorption and open porosity increase with the percentage of biomass bottom ash added. This porosity has already been intuited in previous tests and implies a lower density, as well as a higher thermal and acoustic insulation. In other words, the increase in porosity is not a negative fact if the minimum resistances marked by the regulations are maintained, since it will provide special thermal and acoustic insulation characteristics that are highly desirable in the construction world. It should be borne in mind that, for the life cycle of a building, not only is the energy consumption of the materials used in its manufacture assessed, but also the performance of the building in service. Better insulation qualities directly imply lower energy consumption to keep the temperature of the building constant, and consequently lower CO_2_ emissions over the life of the building.

On the other hand, the apparent density of the ceramics decreases with the increase in the percentage of biomass bottom ash. This parameter is intrinsically related to the previous tests and implies a lower resistance to simple compression, as will be observed in the resistance test. However, the particularities of lower density and higher porosity should not be underestimated. A lower density associated with an acceptable mechanical resistance would also imply obtaining a lighter product that would not overload the weight of the building structure.

The compression strength test (UNE-EN 772-1 standard) of the different families of samples reflects how the family formed only with the clay and without the addition of biomass bottom ash (0A10C) has a compression strength of 155.52 ± 5.60 MPa. This strength is mainly due to the quality of the clay used and the conforming process with adequate humidity, as well as the sintering process. However, it should be noted that these results are in no way comparable to the strength of a brick made from the same material (even with the same forming process), as the brick has different voids and forms that impair compressive strength. At the same time, it is observed that, for the rest of the families conformed, the compression strength decreases progressively, which signifies that the increase in the percentage of addition of biomass bottom ash harms the strength. However, the values obtained are in all cases above the minimum 10 MPa set by the European standard for ceramic materials for bricks. Therefore, all families of samples are suitable for use. This compressive strength value is predicted by the above tests and implies a good behavior of the incorporation of biomass bottom ashes in brick ceramics, since, even if its strength decreases, a by-product is incorporated without any cost and a sustainable material is created. In addition, it is quite likely that the thermal and acoustic insulation in these materials will be increased with respect to a traditional ceramic, since density decreases and porosity increases.

**Figure 3:**
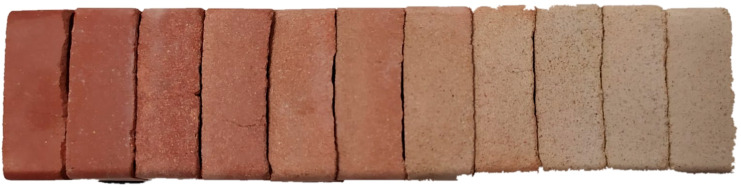
The color of the different families of samples composed of clay and biomass bottom ashes. 100% clay (0A10C) on the left; 100% biomass bottom ash (10A0C) right.

Finally, the color of the different families of samples was studied. Color is an essential aesthetic property for the market. However, its suitability is obviously very subjective, so it was only determined quantitatively. The color was determined with the colorimeter by determining the Red, Green and Blue coordinates of the material, so it is easily reproducible by graphic means. The images of the samples of each family are shown in Figure 3, from the family with 100% clay (0A10C), on the left, to the family with 100% biomass bottom ash (10A0C), on the right.

As can be seen in Figure 3, the increase in the percentage of biomass bottom ash in the ceramic material progressively provides a whitish color. The variation in the color of the samples is due to the chemical composition of each group, since the forming and sintering process is the same for all the groups of samples. The progressive addition of biomass bottom ash causes the percentage of silica to increase, as well as that of calcium oxide, and the percentage of iron oxide to decrease. Therefore, the first family without addition of biomass bottom ash has reddish colors due to iron oxide that, with the addition of biomass bottom ash, tends to be whitish in color due to the increase of silica and calcium oxide. The 10A0C family sample represents the color of the ceramic made up solely of biomass bottom ash and with a low percentage of iron oxide. In line with the above, and in order to objectively determine the color of each group of samples so that they can be recorded and reproduced by graphic means, the color coordinates were measured with the colorimeter. The color coordinates, based on the primary colors, of each of the families mentioned are detailed in Table 6.

The color coordinates of the different groups of samples are another characteristic of the material, not limited by the regulations but by the quality controls of the industry. It is usual that a ceramic material incorporating waste is not accepted by the industry because of the color it reflects, even if it has adequate physical and mechanical characteristics. The quality criteria established by the producing companies have maximum permissible variations in the color of the final material, and therefore the addition of waste that varies sharply in color is rejected. In this case, it can be seen that the variation in color is gradual and toward whitish colors, which is important and easy to market.

As can be seen from the results of the various tests, the use of biomass bottom ash in ceramic materials for brick manufacture involves a variation in physical and color properties. However, the results of none of the families have been rejected according to the European regulations, so they could be manufactured and marketed. In addition, the variations in the abovementioned properties lead to special characteristics of the final product that should be evaluated for specific purposes.

### 3.3. Leaching Tests of the Different Families of Samples

The leaching tests for the different families of samples made up of clay and biomass bottom ashes reflect the retention of the contaminating elements. In this way, it was possible to evaluate the suitability of the groups of samples for a double purpose. On the one hand, the incorporation of a waste that is not used and has a very low economic cost; on the other hand, the retention of contaminating elements that could be leached from the biomass bottom ashes in contact with water in the event that they were deposited in a landfill. In order to evaluate the retention of these contaminating elements, the UNE 772-5 standard was followed, as detailed in the methodology.

**Figure 4:**
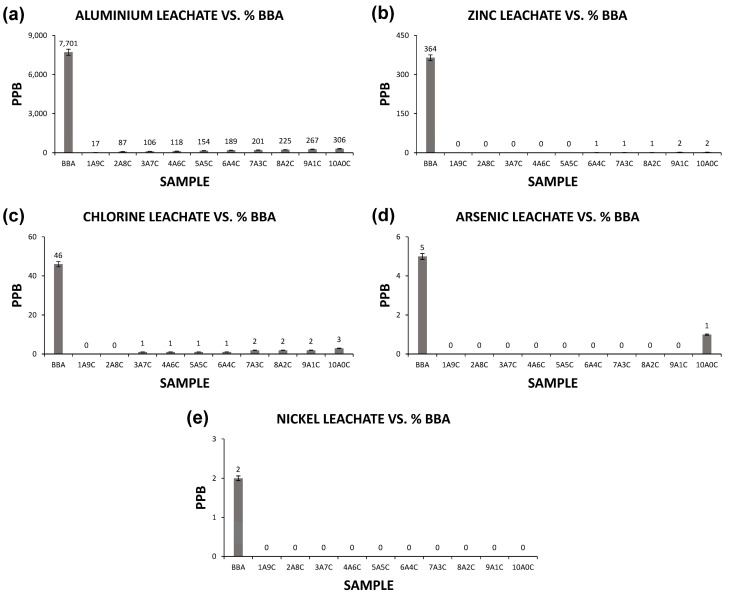
Graphs of the leachates of different chemical elements in the families of ceramic samples and in the unaltered sample of biomass bottom ash. (**a**) aluminium leachates, (**b**) zinc leachates, (**c**) chlorine leachates, (**d**) arsenic leachates and (**e**) nickel leachates.

To this end, the leachate of the different families of samples formed was analyzed and compared with the sample of unaltered biomass bottom ash. The leachate extracted from the different samples was studied quantitatively through the Inductively Coupled Plasm Mass Spectrometry. The results of the commented leachate tests for the chemical elements aluminum, zinc, chlorine, arsenic and nickel are detailed in Figure 4.

As can be seen in Figure 4, there is a significant reduction in the leaching of the chemicals analyzed. The first column of each graph of the leachate elements corresponds to the sample of unaltered biomass bottom ash, and the successive columns to the ceramics formed with increasing percentages of biomass bottom ash. It can be seen how, in the chemical elements aluminum, zinc, chlorine, nickel and arsenic, a drastic reduction of the leachates is produced, retaining these contaminating elements in the ceramic matrix. It should be taken into account that the uncontrolled deposition of the biomass bottom ashes studied can lead to the contamination of surface and groundwater by the presence of the chemical elements analyzed. Aluminum, for example, although not considered a heavy metal, can cause diseases associated with the nervous system. However, aluminum oxide is usually incorporated into ceramics, to improve the strength and hardness of the ceramic material. Therefore, its use in ceramics not only retains its leaching but also causes special characteristics to the final material. The same happens with zinc, when used in ceramics to control linear shrinkage and decrease crack formation, and nickel, which is easily incorporated, as it is a heavy metal. Chlorine, on the other hand, is easily eliminated in the sintering process and is not considered a problem, given its low proportion in the biomass bottom ash. Special mention should be made of the arsenic element, which is so controlled by environmental regulations due to various problems of poisoning by groundwater containing high percentages of the same and which is currently a problem in water purification. With regard to this element, it can be seen that its retention, as it represents a reduced percentage in the ashes, is almost complete, with the maximum proportion of leaching in ceramics being 1 ppb. This value is well below the maximums set by European standards for brick ceramics. 

**Figure 5:**
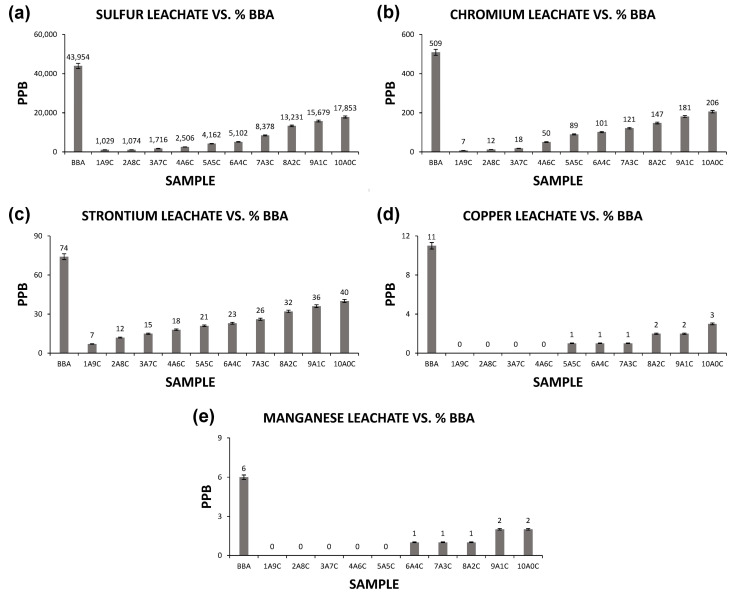
Graphs of the leachates of different chemical elements in the families of ceramic samples and in the unaltered sample of biomass bottom ash. (**a**) sulfur leachates, (**b**) chromium leachates, (**c**) strontium leachates, (**d**) copper leachates and (**e**) manganese leachates.

The results of the leaching tests of the chemical elements sulfur, chromium, strontium, copper and manganese are shown in Figure 5.

Chemical elements such as sulfur, chromium, strontium, copper and manganese are retained in the ceramic matrix but not completely, as can be seen graphically in Figure 5. Therefore, leachates of these elements appear in up to 50% of the leachate from the biomass bottom ash sample of the unaltered sample, in the worst case. This fact is due to the formation temperature of brick ceramics of 950 ± 10 °C; this temperature does not provide that these pollutants are totally combined, so only a part of them is retained. Temperatures higher than the one mentioned would cause the complete retention in ceramic matrix; however, the price and quality of the product would differ from those studied.

However, both copper and chromium are used as stains for ceramics and glazes, and both heavy metals are easily retained in the ceramic matrix. Therefore, the leachates produced from both elements are lower than those marked by the regulations on the subject. At the same time, strontium and manganese are used as fluxes in ceramic materials at high temperatures. However, a higher temperature is required in the sintering process for the total retention of the elements. Finally, sulfur is one of the most harmful elements for the ceramic material. Temperatures higher than the sintering temperature of the bricks would cause a complete crystallization of the element in its matrix and a total retention of the leaching of the element, as it represents a very low percentage in the biomass bottom ash. However, the leachates in the ceramics formed at the determined temperature do not cause problems with respect to the element sulfur.

It must be taken into account that, even if the complete retention of the mentioned elements in the ceramic matrix does not occur, the leachates presented by the different families of samples are below the maximums contemplated by the European regulations for this type of material. 

Therefore, the results obtained are acceptable, and it would not be a limitation according to this test to conform bricks with high percentages of incorporation of biomass bottom ash.

## 4. Conclusions

Once the different tests mentioned in the methodology have been carried out, a series of partial conclusions are obtained which are detailed here and will serve to shape the final conclusion of the work. The importance of using a waste from the electricity generation industry that is not currently used, such as biomass bottom ash, should be highlighted. Its use in ceramic materials for bricks produces an important reduction in the extraction of virgin materials and a lower economic and environmental cost of the final material, as well as a reduction in the deposition of this waste in landfills. In short, a lower environmental impact. The partial conclusions reached are as follows:

- The physical–chemical characterization of the clay and the biomass bottom ashes reflected a compatibility between both materials. This fact is essential to achieve a homogenization of both materials and therefore to obtain a good-quality final material. The particle density of the clay and biomass bottom ashes is 2.44 and 2.85 g/cm^3^, respectively, being quite similar between them. Plasticity is zero for biomass bottom ashes, unlike clays, due mainly to their cementitious composition. Chemical analyses showed a very low percentage of carbon to be expected for both materials due to their origin and formation process. However, X-ray fluorescence reflected some pollutants elements, such as sulfur and arsenic, in the biomass bottom ashes that have been controlled in the leachates of the ceramic materials.
- The physical tests carried out on the different groups of samples formed with increasing percentages of biomass bottom ashes and clay showed similar results of weight loss and decreasing percentages of linear shrinkage after sintering. However, the linear shrinkage was below 8%, so that it be considered a good-quality ceramic for bricks. The values obtained in capillary water absorption range from 1544 to 3054 g/m^2^min. This parameter increases in relation to the increase in the percentage of addition of biomass bottom ash. However, capillary water absorption presented is lower than 4500 g/m^2^min, being this value limiting in order not to affect the durability of the ceramic materials. As for the cold-water absorption, the boiling-water absorption and the open porosity increase as the percentage of biomass bottom ashes increases. This fact is directly related to the compressive strength and in turn to the thermal and acoustic insulation of the conformed ceramic. On the other hand, the bulk density decreases with the increase in the percentage of biomass bottom ash, which is interesting for the manufacture of light materials.
- All the families conformed comply with the limitations of compressive strength. Even the most unfavorable case, which was made only with biomass bottom ashes, had a resistance of around 21 MPa, much higher than the 10 MPa set by European standards.
- The colors of the different groups of samples conformed were measured and quantified with the colorimeter. These groups presented a range of colors, from the traditional ceramic to more whitish colors, with the increase of the percentage of biomass bottom ash.
- The sample of the unaltered material contained chlorine, aluminum, zinc, nickel and arsenic, achieving a drastic reduction in its leachate, even in the families of samples with highest percentage of by-product used. Other elements present in the unaltered material, such as sulfur, chrome, strontium, copper and manganese have been reduced to a lesser extent, mainly due to the sintering temperature of this type of brick ceramics. However, the leachates produced in all the families of samples made and for all the chemical elements detailed are within the acceptable limits of European regulations.

On the basis of the partial conclusions, it can be deduced that it is feasible to use biomass bottom ashes from the combustion of olive pruning and vegetable fat wastes in the ceramics for the manufacture of bricks and up to very high percentages of incorporation.

It is therefore an alternative that provides a fairly adequate solution to the deposition of biomass bottom ash in landfill and possible environmental pollution, obtaining a material with good characteristics and resistant and even with low density and different colors. Moreover, it opens a field in the study of ceramic materials for construction with this waste, not only for the manufacture of bricks, which is the detailed option in which it is easy to incorporate without major modifications and consumes large amounts of raw material, but also in ceramics at higher temperatures. The chemical composition of the biomass bottom ash can create a ceramic material at higher temperatures, with probably very interesting characteristics and with complete retention of the pollutants. It is also worth studying the thermal and acoustic conductivity of ceramics with biomass ashes and bottom, since their incorporation reflected a higher porosity, so it is deductible the good behavior of the material as an insulator. 

In this study, it was possible to achieve, on the basis of detailed conclusions, a sustainable ceramic with lower economic costs, with less environmental impact, with the reuse of industrial waste and within the so-called circular economy.

## Figures and Tables

**Table 1:** Conformed ceramics families with combined percentages of clay and biomass bottom ash.

Samples Groups	Clay, %	BBA, %
0A10C	100	0
1A9C	90	10
2A8C	80	20
3A7C	70	30
4A6C	60	40
5A5C	50	50
6A4C	40	60
7A3C	30	70
8A2C	20	80
9A1C	10	90
10A0C	0	100

**Table 2:** Liquid limit, plastic limit and plasticity index for the clay used.

Test	Value, %
Liquid Limit	38.5 ± 1.7
Plastic Limit	22.1 ± 1.0
Plasticity Index	16.4 ± 0.8

**Table 3:** Elemental analysis carbon, hydrogen, nitrogen and sulfur for clay and biomass bottom ash.

Samples	Nitrogen, %	Carbon, %	Hydrogen, %	Sulfur, %
Clay	0.04 ± 0.00	1.16 ± 0.05	0.65 ± 0.02	0.00 ± 0.00
BBA	0.00 ± 0.00	2.56 ± 0.15	0.63 ± 0.04	0.00 ± 0.00

**Table 4:** Loss on ignition of clay and biomass bottom ash.

Sample	Loss on Ignition, %
Clay	7.90 ± 0.35
BBA	10.35 ± 0.59

**Table 5:** X-ray fluorescence of the clay and biomass bottom ash samples.

Compound	Clay, WT%	BBA, WT%
SiO_2_	52.62 ± 0.25	26.21 ± 0.22
Al_2_O_3_	17.83 ± 0.19	4.54 ± 0.10
Fe_2_O_3_	7.84 ± 0.13	1.97 ± 0.07
K_2_O	5.63 ± 0.12	16.47 ± 0.19
MgO	3.44 ± 0.09	7.58 ± 0.13
CaO	3.19 ± 0.09	25.29 ± 0.22
TiO_2_	0.769 ± 0.038	0.240 ± 0.012
Na_2_O	0.165 ± 0.015	0.274 ± 0.024
P_2_O_5_	0.154 ± 0.008	6.03 ± 0.12
MnO	0.154 ± 0.008	0.1010 ± 0.0050
ZrO_2_	0.0379 ± 0.0049	0.0179 ± 0.0036
V_2_O_5_	0.0357 ± 0.0031	-
SrO	0.0344 ± 0.0036	0.1340 ± 0.0067
RuO_4_	0.0318 ± 0.0021	0.0238 ± 0.0015
Rb_2_O	0.0273 ± 0.0048	-
PdO	0.0273 ± 0.0040	0.0184 ± 0.0027
S	0.0247 ± 0.0013	-
SO_3_	-	0.420 ± 0.021
NiO	0.0233 ± 0.0020	0.0277 ± 0.0015
PtO_2_	0.0184 ± 0.0039	-
Cr_2_O_3_	0.0164 ± 0.0023	0.0137 ± 0.0018
Cl	0.0095 ± 0.0008	0.1750 ± 0.0087
Co_3_O_4_	0.0078 ± 0.0023	-
MoO_3_	0.0063 ± 0.0018	0.0070 ± 0.0014
ZnO	0.0062 ± 0.0027	0.0099 ± 0.0021
As_2_O_3_	-	0.0742 ± 0.0150

**Table 6:** Red, Green and Blue coordinates of the color of the different families of samples conformed with clay and biomass bottom ashes.

Samples Groups	Red	Green	Blue
0A10C	341 ± 15	171 ± 5	117 ± 6
1A9C	306 ± 10	164 ± 7	114 ± 4
2A8C	340 ± 14	197 ± 7	142 ± 5
3A7C	293 ± 14	166 ± 6	119 ± 6
4A6C	287 ± 13	173 ± 8	126 ± 7
5A5C	315 ± 19	207 ± 9	158 ± 5
6A4C	358 ± 17	253 ± 9	197 ± 9
7A3C	384 ± 20	278 ± 11	213 ± 9
8A2C	393 ± 18	320 ± 12	262 ± 14
9A1C	415 ± 15	347 ± 10	285 ± 12
10A0C	361 ± 13	291 ± 16	230 ± 12
